# Antagonistic effects of *Talaromyces muroii* TM28 against Fusarium crown rot of wheat caused by *Fusarium pseudograminearum*

**DOI:** 10.3389/fmicb.2023.1292885

**Published:** 2024-01-03

**Authors:** Han Yang, Shuning Cui, Yanli Wei, Hongmei Li, Jindong Hu, Kai Yang, Yuanzheng Wu, Zhongjuan Zhao, Jishun Li, Yilian Wang, Hetong Yang

**Affiliations:** Ecology Institute of Qilu University of Technology (Shandong Academy of Sciences), Jinan, China

**Keywords:** Fusarium crown rot, *Fusarium pseudograminearum*, *Talaromyces muroii*, antagonistic effects, transcriptome

## Abstract

Fusarium crown rot (FCR) caused by *Fusarium pseudograminearum* is a serious threat to wheat production worldwide. This study aimed to assess the effects of *Talaromyces muroii* strain TM28 isolated from root of *Panax quinquefolius* against *F. pseudograminearum*. The strain of TM28 inhibited mycelial growth of *F. pseudograminearum* by 87.8% at 72 h, its cell free fermentation filtrate had a strong antagonistic effect on mycelial growth and conidial germination of *F. pseudograminearum* by destroying the integrity of the cell membrane. In the greenhouse, TM28 significantly increased wheat fresh weight and height in the presence of pathogen *Fp*, it enhanced the antioxidant defense activity and ameliorated the negative effects of *F. pseudograminearum*, including disease severity and pathogen abundance in the rhizosphere soil, root and stem base of wheat. RNA-seq of *F. pseudograminearum* under TM28 antagonistic revealed 2,823 differentially expressed genes (DEGs). Most DEGs related to cell wall and cell membrane synthesis were significantly downregulated, the culture filtrate of TM28 affected the pathways of fatty acid synthesis, steroid synthesis, glycolysis, and the citrate acid cycle. *T. muroii* TM28 appears to have significant potential in controlling wheat Fusarium crown rot caused by *F. pseudograminearum.*

## 1 Introduction

Fusarium crown rot (FCR), which is a soil-borne disease caused by various *Fusarium* species, is one of the most significant wheat diseases in many countries (Kazan and Gardiner, 2018), The incidence of FCR disease showed a rapid increase year by year, particularly under the adoption of wheat-maize crop rotation and straw return cropping patterns. The dominant pathogen causing FCR in wheat varies in different regions of Central Asia (Bozoglu et al., 2022; Ozer et al., 2023), Australia (Saad et al., 2021, 2022; Buster et al., 2023), Africa (Hadjout et al., 2022; Mawcha et al., 2022; Theron et al., 2023), the Middle East (Minati, 2020; Ozer et al., 2020) and North America (Hagerty et al., 2021; Shrestha et al., 2021), *F. pseudograminearum* and *F. graminearum* are the main pathogens. FCR caused by *F. pseudograminearum* (*Fp*) was first reported in Henan province, China (Li et al., 2012), and has since become the dominant pathogen in the Huanghuai Plain. It not only causes severe yield and economic losses to wheat but also produces toxins and secondary metabolites like DON (Palacios et al., 2017), which also have serious negative impacts on food products and safety.

Even though chemical control can reduce the damage caused by FCR, the long-term use of chemical fungicides will have negative health effects. The most important factor is the gradual increase in pathogen resistance to chemical fungicides (Blyuss et al., 2019). In recent years, a variety of microorganisms with potential antagonistic activity have been identified, which can inhibit the growth of *Fp* and effectively reduce the severity of wheat FCR (Zhao et al., 2022). For instance, *Trichoderma* has been extensively studied in plant disease suppression (Blaszczyk et al., 2017; Hewedy et al., 2020), and can inhibit *Fp* by colonization (Sarrocco et al., 2021), mycoparasitism (Alfiky and Weisskopf, 2021), and rhizosphere competition (Rawat and Tewari, 2011; Anees et al., 2018). *Bacillus* also has antagonistic activities against *Fp*, and produces indoleacetic acid that promotes plant growth and has biocontrol potential (Li et al., 2022; Zhang et al., 2022). In addition, the combined use of *Trichoderma* and *Bacillus* spp. was more effective than acting alone, suggesting a synergistic effect (Zhou et al., 2021). *Pseudomonas* has antagonistic activities against *Fp* by producing antibiotics, such as phenazines and 2,4-diacetylphloroglucinol (Zhang J. B. et al., 2020). *Streptomyces*, a biocontrol microorganism, is widely distributed in plants and soil, and it inhibits *Fp* through antibiosis, interference with space and nutrient availability (Winter et al., 2019; O'Sullivan et al., 2021).

*Talaromyces* spp. was originally considered a genus of *Penicillium*. It was first described by Benjamin in 1955 (Zhang et al., 2021). According to phylogenetic studies, *Penicillium* subgenus *Biverticillium* was transferred to *Talaromyces*. Thus, the genus *Penicillium* has been redefined as *Penicillium sensu stricto* and *Talaromyces* (Chen et al., 2016). *Talaromyces* originated from the ocean and now resides as endophytes in air, soil, decaying food, and healthy plants (Wang and Zhuang, 2022). Studies have shown that *Talaromyces* and its secondary metabolites can exhibit antibacterial, antifungal, and antitumor activities (Rateb and Ebel, 2011), which is of great value for further research (Zhao et al., 2022). *Talaromyces* genomes contain a rich and diverse set of primary and secondary metabolic genes (clusters) encoding a variety of proteases, carbohydrate-active enzymes, fungal cell wall-degrading enzymes, lectins, and secondary metabolite biosynthetic enzymes (Wang et al., 2020). *Talaromyces* spp. can promote plant growth and suppress pathogens in agriculture. *T. flavus* has been widely researched, and several strains with strong antagonistic activities have been isolated and evaluated. Various isolates of *T. flavus* have been shown to promote plant growth and induce resistance in various crop species (Naraghi et al., 2010a). *T. transit* can reduce brown spot disease and dirty panicle diseases in rice (Dethoup et al., 2018), whereas *T. lamosis* can effectively treat rot diseases in cucumbers and tomatoes effectively (Halo et al., 2019).

In a previous study, the TM28 strain of *T. muroii* was isolated from the root of American ginseng (*Panax quinquefolius*) and demonstrated broad antagonistic activity against 12 pathogenic fungi. The objectives of this study were to investigate the inhibitory activity of TM28 on *Fp* mycelial growth and spore germination, determine the mechanism of inhibitory activity using RNA-seq, and study the potential biocontrol activity of TM28 against wheat FCR. This study attempted to elucidate the antagonistic mechanism of *Fp* inhibition by the TM28 strain and provide effective technical support for the prevention and control of FCR in wheat.

## 2 Materials and methods

### 2.1 Fungal material and culture condition

The *Talaromyces muroii* strain TM28 was isolated from the root of American ginseng in Weihai, Shandong Province, which has demonstrated greater antagonistic activities and promoted the growth of cucumber seedlings by producing ACC deaminase (will be presented in another paper). The pathogenic fungus was *F. pseudograminearum* (*Fp*), which was identified and stored at the Environmental Microbiology Laboratory of the Ecology Institute of Shandong Academy of Sciences.

Both *T. muroii* TM28 and *Fp* were cultivated on potato dextrose agar (PDA) medium (200 g L^−1^ potato, 20 g L^−1^ glucose, 15 g L^−1^ agar, 1,000 mL H_2_O) at 28°C. Conidia suspensions of *Fp* were obtained from fermentation medium (maize powder 20 g L^−1^, soybean powder, 10 g L^−1^, K_2_HPO_4_ 5 g L^−1^, KH_2_PO_4_ 2 g L^−1^, glucose 5 g L^−1^) and cultured at 28°C with shaking at 180 rpm for 7 days to produce large number of spores (Cho et al., 2013). The sterile fermentation filtrate of TM28 was obtained from potato dextrose broth (PDB) cultured at 28°C with shaking at 180 rpm for 20 days to produce a mess of metabolite, and centrifuged at 8,000 rpm for 10 min. The supernatant was filtered through a 0.22 μm membrane and stored at 4°C for future experiments.

### 2.2 Effects of *Talaromyces muroii* TM28 on hyphae of *Fusarium pseudograminearum*

The dual culture method (Pellan et al., 2020) was used to assay the effect of TM28 on mycelial growth of *Fp*. TM28 and *Fp* mycelial discs of 5 mm in diameter were inoculated at two relative points 2 cm from the center of the PDA plate; only *Fp* was used as a control. All treatments were carried out in triplicate and incubated at 28°C. On days 3 and 6, the distance between the center and outer edge of *Fp* was measured and the inhibition rate was calculated. The hyphal morphology of *Fp* was examined under a light microscope and compared to that of the control.

To assay the effect of TM28 fermentation filtrate on hyphal growth of *Fp*, the filtrate was mixed with PDA medium to obtain plates with 4, 10, and 20% (v/v) content, respectively. Then, a 5 mm diameter mycelial disc of *Fp* from 3-day-old PDA was inoculated on the center of the plates and incubated at 28°C, a disc grown on PDA without filtrate was used as control. Every treatment was carried out in triplicate. The colony diameters were measured after culturing for 24 h, 48 h, and 72 h, respectively (Akpinar et al., 2021). At the same time, the effect of the TM28 filtrate on hyphae of *Fp* growth under liquid culture conditions was analyzed. One milliliter of *Fp* spore suspension (10^7^ CFU mL^−1^) was added to a conical flask with 50 mL PDB medium, cultured at 28°C with shaking at 150 rpm for 12 h, 2.5 mL of TM28 fermentation filtrate was added and cultured for another 48 h. The hyphae of *Fp* were collected in a suction flask and dried in an oven at 65°C until constant weight (Darsih et al., 2018).

### 2.3 Effects of *Talaromyces muroii* TM28 on conidial germination of *Fusarium pseudograminearum*

The fermentation filtrate of TM28 was diluted 5 ×, 10 ×, 25 ×, and 50 × with sterile PDB medium, and 1 mL was added to 100 uL *Fp* conidial suspension, cultured at 26°C in the dark, and conidial germination was observed under a light microscope every 2 h. As controls, equal volumes of sterile PDB broth and *Fp* conidial suspension were also used, with three replicates for each treatment. Spores were deemed to have germinated if the germ tube exceeded 50% of the spore’s length, and 150–200 spores were counted (Piermann et al., 2023).

### 2.4 Effects of *Talaromyces muroii* TM28 on cell membrane permeability and enzyme activities of *Fusarium pseudograminearum*

One milliliter of *Fp* spore suspension (10^7^ CFU mL^−1^) was added to a conical flask containing 50 mL PDB medium and incubated at 28°C for 12 h, followed by the addition of 2.5 mL of TM28 fermentation filtrate and incubation for an additional 48 h. As a control, the same volume of sterile PDB broth was added, and three replicates were used in each experiment. Finally, the cultured broth was centrifuged at 8,000 rpm for 5 min, and the electrical conductivity of the supernatant was measured using a pH meter (Mettler Toledo, Germany), which expresses the permeability of the cell membrane, according to the method of Frallicciardi et al. (2022). The crude enzyme solution was prepared from 0.1 g mycelia samples and the enzyme activities were determined using commercial Succinate Dehydrogenase (SDH), hexokinase (HK) and phosphofructokinase (PFK) activity kits according to the manufacturer’s instructions (Beijing Solarbio Science and Technology, Ltd., Beijing, China).

### 2.5 *Talaromyces muroii* TM28 suppression of wheat FCR in the pot experiment

The seeds of wheat varieties Jimai 22 and Jimai 44 were provided by Shandong Luyan Agricultural Co. Ltd. (Jinan, China). These two varieties are the main cultivars in Shandong Province, among which Jimai 44, characterized as a high-gluten variety, exhibits greater sensitivity to Fusarium crown rot caused by *Fp*. The soil for the pot experiment was collected from the natural garden in Jinan with the physicochemical characteristics of organic matter 36.48 mg g^−1^, total N 301.23 mg kg^−1^, available P 46.25 mg kg^−1^, and available K 118.14 mg kg^−1^. The soil was passed through a 20-mesh sieve to eliminate plant debris. The planting buckets were 20 cm high and 10 cm in diameter, filled with 500 g soil and 300 mL *Fp* spore suspension (10^6^ CFU mL^−1^), mixed thoroughly, and pre-cultured at room temperature for three days. The soil without *Fp* addition was set as the blank control. The wheat seeds were surface-sterilized with sodium hypochlorite solution (1% available chlorine) for 2 min, followed by 70% (v/v) ethanol for 30 s. After five consecutive washes in sterile distilled water, the seeds were soaked in a conidial suspension of TM28 (10^8^ CFU mL^−1^) or sterile distilled water for 30 min. After drying under a laminar flow for 1 h, 15 seeds were sown in each bucket. Six replicates were set for each treatment. Wheat seedlings were grown in a greenhouse with a day/night cycle of 15–9 h, 25–16°C, and 55% relative humidity. The wheat seedlings were thinned on the 10th day after germination to maintain 10 seedlings per pot. All the pots were watered at regular intervals.

Seedlings were harvested on the 30th day after sowing. Shoots and roots were collected for height and biomass measurements. Fresh wheat leaves (0.5 g) were ground in liquid nitrogen to test the physiological indices, including chlorophyll content, malonaldehyde (MDA) content, and antioxidant enzyme activities such as superoxide dismutase (SOD), peroxidase (POD), and catalase (CAT), using commercial kits according to the manufacturer instructions (Beijing Solarbio Science and Technology, Ltd., Beijing, China).

According to the length of stem browning under the first leaf sheath, wheat FCR disease was divided into five levels (Ullah et al., 2020): Grade 0: robust plants with no browning symptoms; Grade 1: only the first leaf sheath brown, and the browning area did not exceed 1/2; Grade 3: only the first leaf sheath turned brown, and the browning area exceeded 1/2; Grade 5: the second leaf sheath turned brown; Grade 7: the third leaf sheath turned brown; Level 9: plant death. Using the following formulas, the disease index and control effectiveness were computed.

$\begin{array}{c} \mathrm{Disease index}=100\times \Sigma \left(\mathrm{grade}\times \mathrm{number of infected plants}\right)/\\ \;\left(\mathrm{highest grade}\times \mathrm{total number of investigated plants}\right)\text{.} \end{array}$

$\begin{array}{c} \mathrm{Control efficiency}=\left(\mathrm{disease index of control-disease index of treatment}\right)/\\ \;\mathrm{disease index of control}\times 100\text{.} \end{array}$

### 2.6 Total genomic DNA extraction and quantitative PCR

Rhizosphere soil adhering to the roots was meticulously collected through gentle shaking. The roots and stem bases of the wheat were washed five times in sterile distilled water, ultrasonicated for 30 min, and dried with sterile filter paper. Using the Power Soil DNA Isolation Kit (Qiagen, Germany), total genomic DNA was extracted from 0.25 g soil, roots, and stem base samples according to the manufacturer instructions. The extracted DNA was electrophoresed on 1% agarose gel, and its concentration and purification were measured using a NanoDrop 2000 spectrophotometer (Thermo, United States).

The abundance of *Fp* was determined by quantitative polymerase chain reaction (qPCR) using the TaqMan probe method (Hogg et al., 2007). The probe was labeled with a 6-carboxyfluorescein (6-FAM) fluorescence reporter (5′-6FAM-TGCTTACAACAAGGCTGCC CACCA-TAMRA-3′). *Fp* genomic DNA was used as a template, and PCR amplification was performed using the specific primers FP-TEF1A.2F (CTTCTTTCACCGCTCAGGTC) and FP-TEF1A.2R (CTTGGAGGGAACCATCTTGA). Standard curves were generated using 10-fold serial dilutions of plasmids containing the targeted DNA fragments. The 20 μL qPCR reactions contained 10 μL TaqMan Fast qPCR Master Mix (TaKaRa, Dalian, China), 0.4 μL of each 10 μM forward and reverse primers, 10 μM Probe 0.3 μL, 2.0 μL DNF Buffer, 1.0 μL DNA samples and 5.9 μL sterile and DNA-free water. The two-step qPCR assay was performed at 95°C for 3 min. 95°C 5 s, 60°C 30 s, 40 cycles. All qPCR reactions were conducted in triplicates. The number of *Fp* copies per gram of the sample was calculated using the following equation:

$\begin{array}{l} \mathrm{Number}\;\left(\mathrm{copies}\;g-1\right)\\ =\left(10^{SQ}\times 6.02\times 10^{23}\times 10^{-9}\times V\right)/\left(660\times SD\times m\right)\text{.} \end{array}$

Where SQ is the sample concentration, V is the extracted DNA volume of the sample (μL), m is the biomass of the sample (g), and SD is the base number of the standard plasmid (2,980 bp).

### 2.7 Transcriptome analysis of *Fusarium pseudograminearum* co-culture with *Talaromyces muroii* TM28 fermentation filtrate

One milliliter of *Fp* spore suspension (10^7^ CFU mL ^−1^) was added to a conical flask containing 50 mL PDB medium and incubated at 28°C for 12 h, then 2.5 mL of TM28 fermentation filtrate was added and co-cultured at 28°C with shaking at 150 rpm for 24 h, the same volumes of sterile PDB broth added as the control. Three replicates were used for each treatment. At the end of incubation, washed the mycelia 3 times with cold sterile water and centrifuged at 12,000 × g for 2 min at 4°C. The precipitate was frozen in liquid nitrogen immediately and stored at −80°C until RNA extraction.

Total RNA was isolated from 0.1 g of each of the mycelia of six samples using TRIzol reagent (Invitrogen, United States) according to the manufacturer’s instructions. RNA concentration and quality were determined using a NanoDrop spectrophotometer (Thermo Fisher Scientific). Transcriptome sequencing and analyses were performed by Sangon Biotech Co. Ltd. (Shanghai, China), using the Illumina HiSeq platform. The raw data were quality-controlled using FastQC (version 0.11.2) and Trimmomatic (version 0.36) to produce clean data. The HISAT2 software was used to compare the valid data of the sample with the reference genome of *F. pseudograminearum* Class2-1C^1^ for statistical mapping information. Valid data were compared to the assembled transcriptome sequence, and RSEM (version v1.2.8) was used to determine expression levels. Venn diagrams, heat maps, and cluster analyses were performed based on the results of the difference analysis. DESeq2 (version 1.12.4) was used for gene expression difference analysis, and genes with multiple |FoldChange| ≥ 2 and *q* value <0.05 as significantly differentially expressed genes (DEGs). We used topGO (version 2.24.0) for GO enrichment analysis and cluster Profiler (version 3.0.5) for Kyoto Encyclopedia of Genes and Genomes (KEGG) pathway enrichment analysis.

### 2.8 Quantitative reverse transcription PCR analysis

For reverse transcription-polymerase chain reaction (RT-PCR), first-strand cDNA was reverse-transcribed from the total RNA of *Fp* for RNA-seq using the Evo M-MLV RT Premix kit (AG11607, Accurate Biology, Hunan, China) according to the manufacturer’s instructions. Quantitative RT-PCR (qRT-PCR) was performed using a CFX96 optical real-time detection system (Bio-Rad Laboratories Inc., Hercules, CA, United States). The 20 μL reactions contained 10 μL 2 × Power SYBR® Green PCR Master Mix (TaKaRa, Dalian, China), 0.4 μL of each 10 μM forward and reverse primers, 2.0 μL cDNA samples, and 7.2 μL sterile and DNA-free water. The PCR program was: pre-denaturation at 95°C for 30 s, denaturation at 95°C for 5 s, annealing at 54°C for 30 s, extension at 72°C for 20 s, for 40 cycles. Amplicon dissociation curves were recorded after cycle 40 by heating from 60 to 95°C with a ramp speed of 1.0°C per min. All reactions were performed with at least three technical replicates. *TEF1α* was used as the internal reference gene, and the relative expression was calculated using the 2^-ΔΔt^ method (Schmittgen and Livak, 2008). Twenty DEGs were selected, and primers were designed using Beacon Designer 7 and synthesized by Changsheng Biotechnology Co., Ltd. (Dingguo, Beijing, China). All primers are listed in Supplementary Table S1.

### 2.9 Data analysis

The data were statistically analyzed using one-way ANOVA and Duncan’s method (*p* < 0.05) in SPSS 26.0 statistical software for significance analysis. GraphPadPrism (8.3.0.538) was used to calculate and plot the data.

## 3 Results

### 3.1 Effects of *Talaromyces muroii* TM28 on hyphae of *Fusarium pseudograminearum*

**Figure 1:**
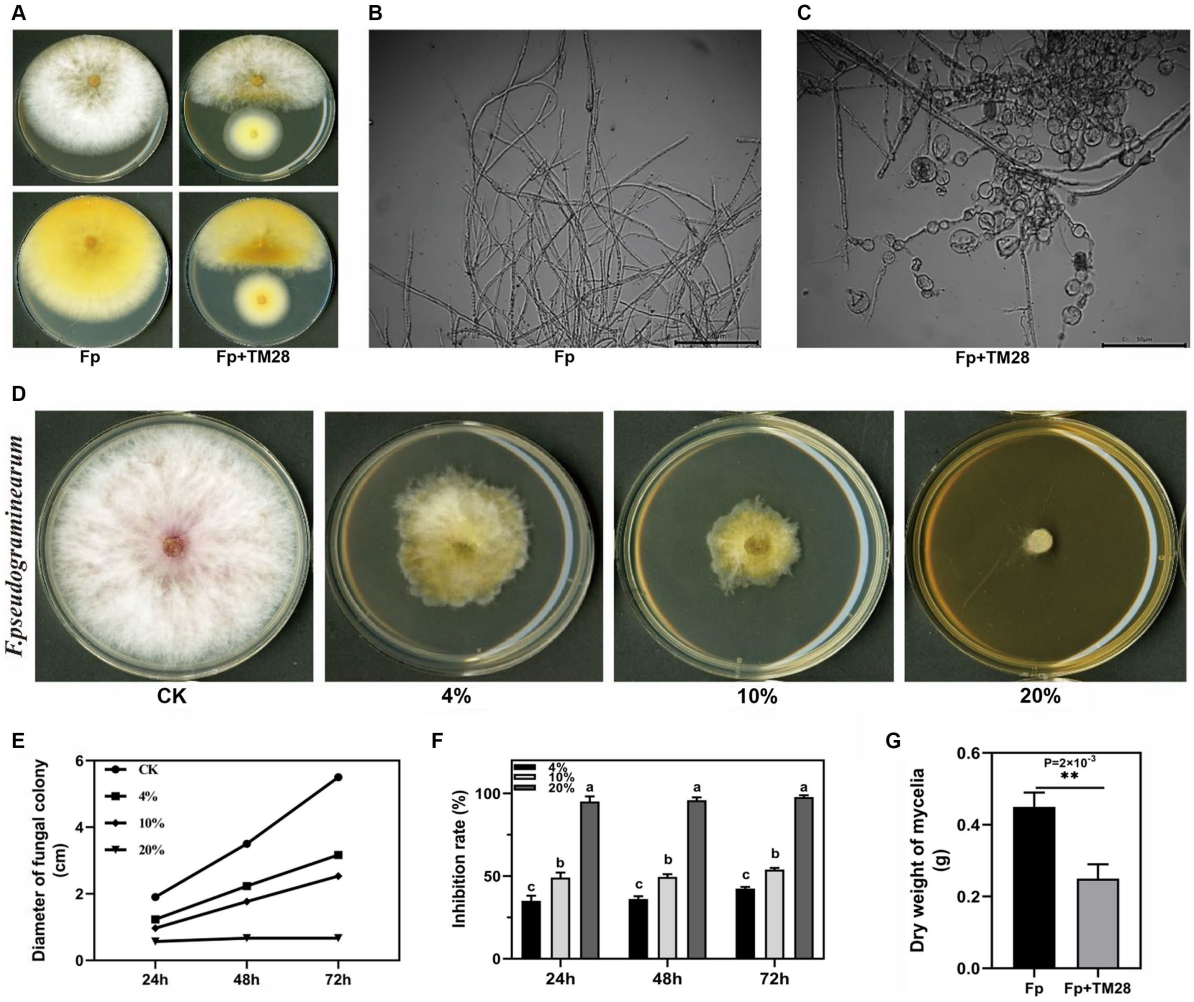
Effect of TM28 on the growth of *F. pseudograminearum.* **(A)** *F. pseudograminearum* growth with *T. muroii* TM28 on PDA. **(B)** Microscopic observation of the hyphae of *F. pseudograminearum* growing without *T.muroii* TM28. **(C)** Microscopic observation of the hyphae of *F. pseudograminearum* growing with *T. muroii* TM28. **(D)** The inhibition activity of different concentrations of *T. muroii* TM28 fermentation filtrate on hyphae growth of *F. pseudograminearum* on PDA plates. **(E)** Effect of different concentrations of *T. muroii* TM28 filtrate on the mycelial diameter. **(F)** The inhibition rate of different concentrations of *T. muroii* TM28 filtrate on the growth of *F. pseudograminearum*. **(G)** Effect of *T. muroii* TM28 filtrate on *F. pseudograminearum* growth in liquid culture. Columns with different lowercase letters indicated significant differences between the compared groups (*p* < 0.05), ** represent significant differences at *p* < 0.01 according to one-way analysis of variance (ANOVA).

After co-culture with TM28, *Fp* was significantly inhibited on the PDA plate, with an inhibition rate of 87.8% at 72 h (*p* < 0.05) (Figure 1A). The hyphae of *Fp* were close to the edge of the TM28 colony atrophied, and pigment production of *Fp* was promoted. Optical microscopy analyses revealed that hyphae in the control group exhibited a normal morphology with more branches, a smooth surface, and uniform thickness (Figure 1B). However, after antagonistic culture with TM28, the morphology of *Fp* hyphae underwent significant changed. Some hyphae were swollen and malformed with short and tangled branches, while others were shriveled and severely vesiculated (Figure 1C).

The TM28 fermentation filtrate also inhibited *Fp* growth, as the concentration of the filtrate increased, the inhibition effect was higher. When the concentration in PDA medium reached 20%, the growth of *Fp* inhibited with dense hyphae, and some hyphae extended into the air (Figure 1D). The diameter of *Fp* in 4 and 10% filtrate content treatment group were 31.67 mm and 25.33 mm at 72 h, (Figure 1E), the inhibition rates were 42.42 and 53.94% compared with the control group, respectively (*p* < 0.05, Figure 1F). The mycelial biomass of the control group was 0.45 g under liquid culture conditions, whereas the mycelial weight was only 0.25 g at 5% TM28 filtrate content, and mycelial growth inhibition was 44.44% (*p* < 0.01, Figure 1G).

### 3.2 Effects of *Talaromyces muroii* TM28 on conidia germination of *Fusarium pseudograminearum*

**Figure 2:**
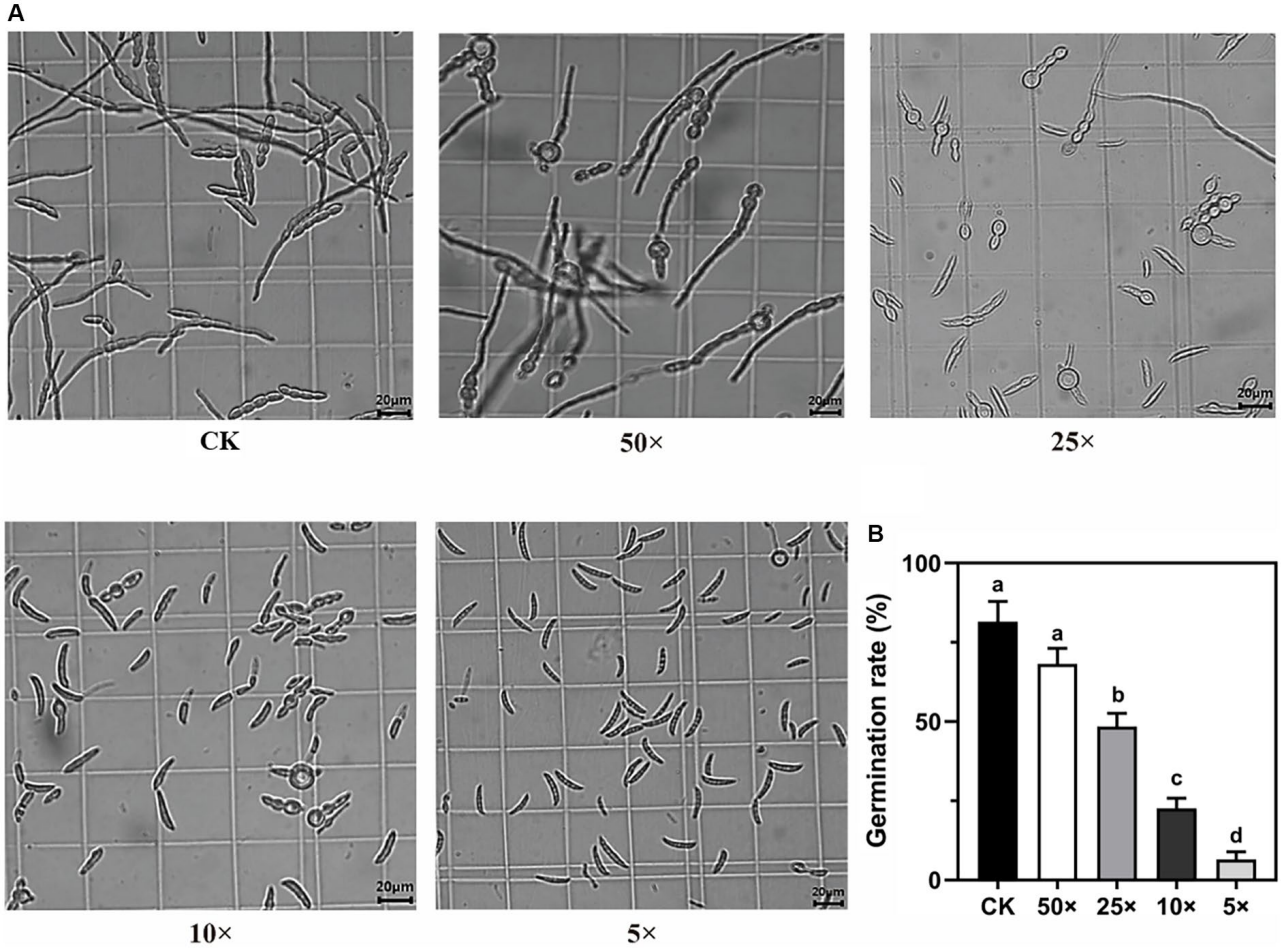
Effect of the fermentation filtrate of TM28 on conidia germination of *F. pseudograminearum.* **(A)** Microscopic observation of the conidia of *F. pseudograminearum* at different fermentation filtrate concentrations. **(B)** Effect of the fermentation filtrate of *T. muroii* TM28 on conidia germination rate of *F. pseudograminearum* at 12 h. Columns with different lowercase letters indicated significant differences between the compared groups (*p* < 0.05).

The germination of *Fp* conidia was significantly inhibited by the TM28 fermentation filtrate, and the rate of inhibition increased with increasing filtrate concentration (Figure 2A). After 12 h incubation, conidia of *Fp* vigorously germinated and formed an interwoven network in the control group (Figure 2A), with a germination rate of 81.58% (Figure 2B). Compared to the control, 50 × dilution of the TM28 fermentation filtrate had no significant effect on *Fp* spore germination. The germination rates of *Fp* conidia treated with 25 × and 10 × dilutions were only 47.57 and 23.73%, respectively, and the morphology of germinated conidia swollen and deformed within the germ tube was shorter than that of control and maintained a bubble-like appearance (Figure 2A). Germination of *Fp* spores was nearly completely inhibited in the 5 × dilution treatment, with no expansion or production of germ tubes. The inhibition rates of *Fp* conidia co-cultured with 25 ×, 10 ×, and 5 × dilutions of TM28 filtrate were 52.43, 76.27, and 93.42%, respectively (*p* < 0.05, Figure 2B).

### 3.3 Effects of *Talaromyces muroii* TM28 on cell membrane permeability and enzyme activities of *Fusarium pseudograminearum*

**Figure 3:**
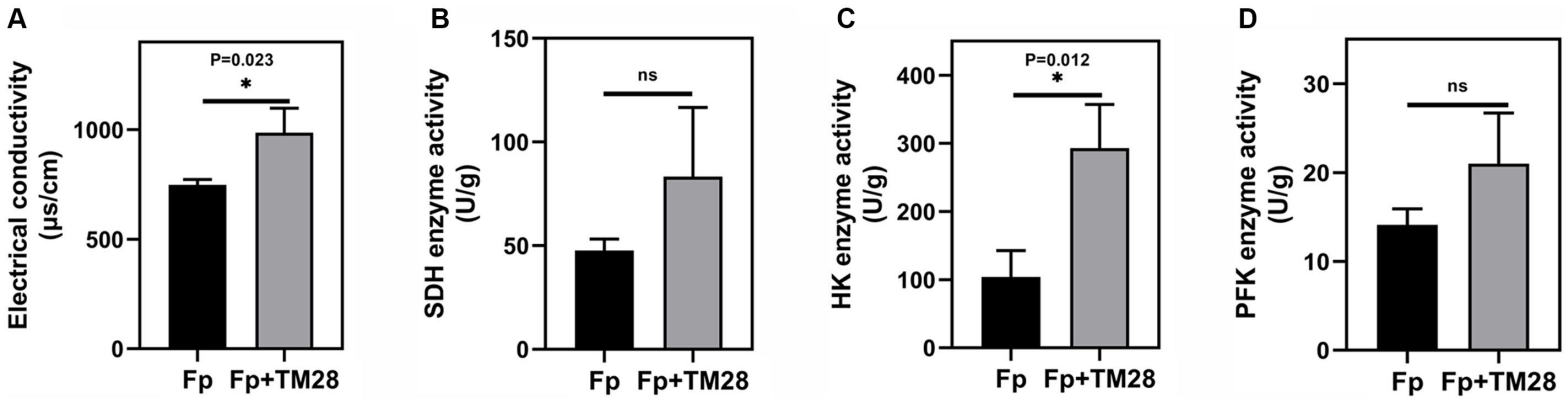
Effects of the fermentation filtrate of TM28 on the cell membrane permeability and enzyme activities of *F. pseudograminearum.* **(A)** Electrical conductivity; **(B)** Succinate Dehydrogenase (SDH) activity; **(C)** Hexokinase (HK) activity; **(D)** Phosphofructokinase (PFK) activity.

In this study, the effect of TM28 fermentation filtrate on the permeability of the *Fp* membrane was determined by measuring the variation in electrical conductivity. The results showed that the electrical conductivity of *Fp* fermentation broth significantly increased in the TM28 treated group compared to that in the control (p < 0.05, Figure 3A), presumably due to the leakage of intracellular material from *Fp* cells as a result of damage to the cell membrane. After treatment with TM28, the SDH activity of *Fp* increased from 47.66 U mg ^−1^ to 87 U mg ^−1^ compared to that of the control, but the difference was not significant (Figure 3B). For HK and PFK, which were the key rate-limiting enzymes in the glycolytic and TCA pathway (Fan et al., 2022), we observed that HK activity was significantly increased from 103.88 U g ^−1^ to 293.09 U g ^−1^ after treated with TM28 (*p* < 0.05, Figure 3C). PFK activity was increased from 14.1 U g ^−1^ to 21.0 U g ^−1^ without significant difference (Figure 3D).

### 3.4 TM28 inhibition of FCR in greenhouse

**Figure 4:**
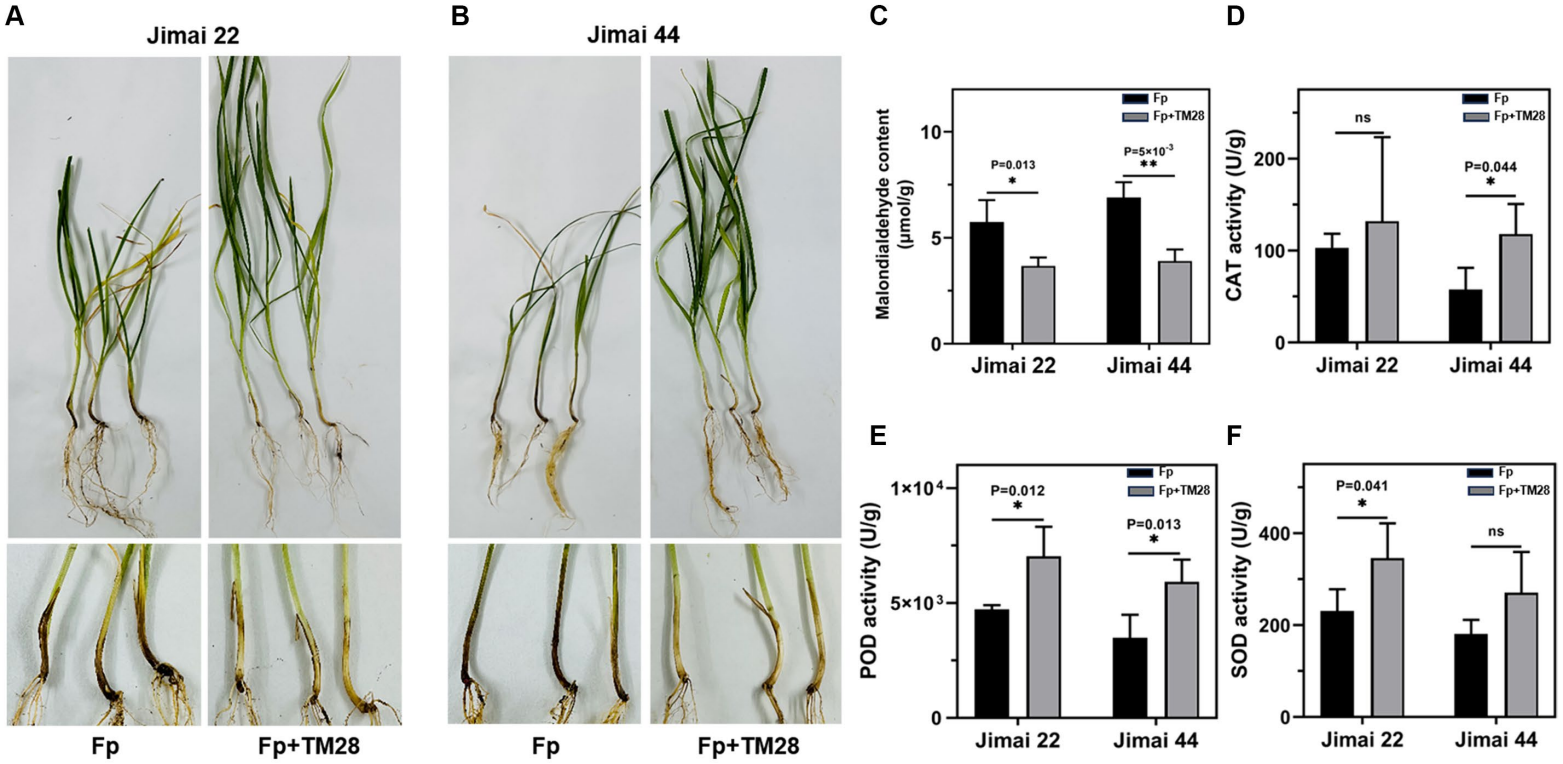
Effect of treatment with TM28 on the growth of wheat plants in the greenhouse. **(A)** Seedlings of Jimai 22, Fp indicated seeds treated with sterilized water and *F. pseudograminearum* inoculated into the soil, Fp + TM28 indicated seeds treated with *T. muroii* TM28 and *F. pseudograminearum* inoculated into the soil. **(B)** Seedlings of Jimai 44, Fp indicated seeds treated with sterilized water and *F. pseudograminearum* inoculated into the soil, Fp + TM28 indicated seeds treated with *T. muroii* TM28 and *F. pseudograminearum* inoculated into the soil. **(C)** The Malondialdehyde (MDA) content in wheat leaves; **(D)** The activity of catalase (CAT) of wheat leaves; **(E)** The activity of peroxidase (POD) of wheat leaves; **(F)** The activity of superoxide dismutase (SOD) of wheat leaves. The results are expressed as the mean ± SD of six biological replicates. Bars indicate standard deviation, asterisks indicate significant differences between samples (*p < 0.05; **p < 0.01) according to Tukey’s test.

To further investigate the biocontrol activity of TM28 against *Fp*, a pot experiment was conducted in a greenhouse. The results showed that wheat seedlings inoculated with *Fp* showed FCR symptoms of dwarf, stunting, black or brown leaf sheaths, and root rot (Figures 4A,B). The two varieties of wheat had different degrees of infection with the disease index of Jimai 22 was 81.38, while that of Jimai 44 was 85.56. For TM28 treatment plus *Fp* inoculation, the disease index was significantly less than with *Fp* alone, with the control effects of 58.49 and 51.69% for Jimai 22 and Jimai 44, respectively (Table 1).

**Table 1:** Effect of TM28 on wheat seedling growth and FCR levels.

	Treatment	Plant Height (cm)	Shoot Fresh Weight (g)	Chlorophyll Content (mg/g)	Disease Index	Relative Control Effect (%)
Jimai 22	CK	27.22 ± 0.62 ab	0.86 ± 0.04 ab	0.15 ± 0.04 a		
	TM28	28.72 ± 0.35 a	0.92 ± 0.11 a	0.16 ± 0.05 a		
	Fp	23.78 ± 1.71 c	0.64 ± 0.16 c	0.14 ± 0.03 a	81.38 ± 5.97 a	
	Fp + TM28	26.49 ± 0.68 b	0.73 ± 0.08 bc	0.15 ± 0.04 a	33.78 ± 4.82 b	58.49
Jimai 44	CK	25.98 ± 0.86 a	0.70 ± 0.04 ab	0.15 ± 0.01 a		
	TM28	27.05 ± 1.39 a	0.76 ± 0.09 a	0.17 ± 0.02 a		
	Fp	22.97 ± 1.18 b	0.57 ± 0.03 b	0.15 ± 0.01 a	85.56 ± 5.16 a	
	Fp + TM28	25.10 ± 0.47 a	0.63 ± 0.17 ab	0.16 ± 0.03 a	41.33 ± 7.95 b	51.69

CK indicated seeds treated with sterilized water without *F. pseudograminearum* inoculated into the soil, TM28 indicated seeds treated with TM28 and without *F. pseudograminearum* inoculated into the soil, Fp indicated seeds treated with sterilized water and *F. pseudograminearum* inoculated into the soil, Fp + TM28 indicated seeds treated with *T. muroii* TM28 and *F. pseudograminearum* inoculated into the soil. Data in the table are mean ± standard deviation (SD). Means with a different lowercase letter indicated significant differences (*p* < 0.05) between the compared groups using one-way analysis of variance (ANOVA).

Thirty days after sowing, there was no significant difference in plant fresh weight and plant height of TM28 treated wheat as compared to blank control in the absence of pathogen *Fp* (Table 1). While TM28 treatment plus *Fp* inoculation significantly increased the plant height and fresh weight of both wheat varieties. There were no significant differences in chlorophyll content among all treatments (Table 1).

**Figure 5:**
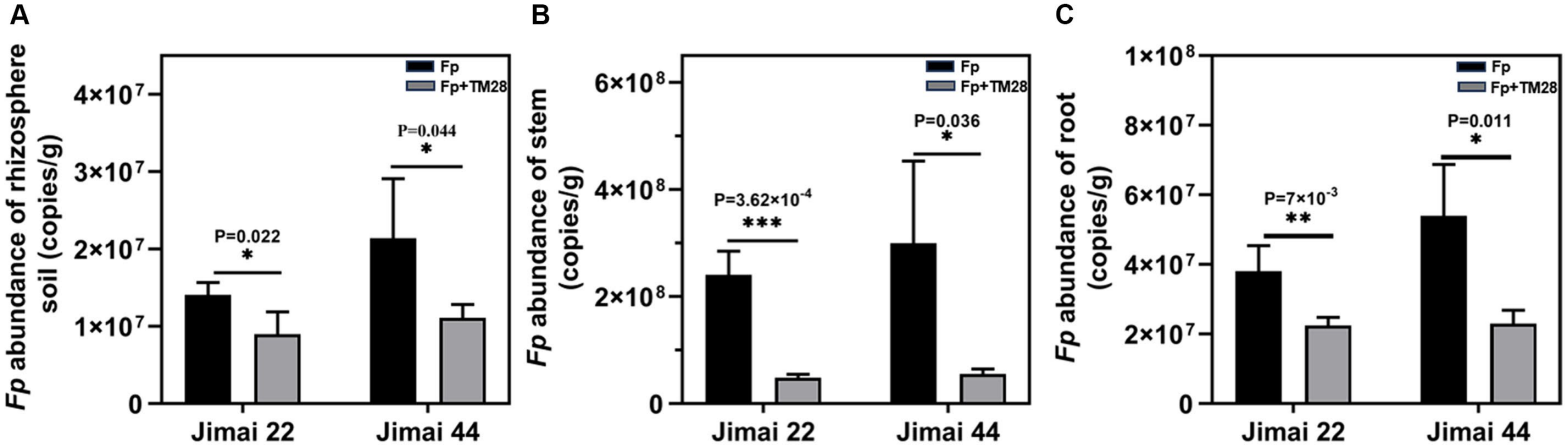
Effect of TM28 on the abundance of *F. pseudograminearum* in different parts of wheat. **(A)** The abundance of *F. pseudograminearum* in rhizosphere soil; **(B)** The abundance of *F. pseudograminearum* in wheat stem; **(C)** The abundance of *F. pseudograminearum* in wheat root. Jimai 22 and Jimai 44 are different varieties of wheat. The results are expressed as the mean ± SD of six biological replicates. Bars indicate standard deviation; asterisks indicate significant differences between samples (*p < 0.05; **p < 0.01; ***p < 0.001) according to Tukey’s test.

TM28 treatment significantly decreased the MDA content of wheat seedlings compared to *Fp* infected plants (Figure 4C). In contrast, the activities of CAT and POD in Jimai 44 were significantly higher than those in the control (Figures 4D–F). In the case of Jimai 22, TM28-inoculated wheat plants exhibited a significant increase in the activities of POD and SOD compared to pathogen-infected plants, while there was no significant difference in the activity of CAT (Figures 4D–F). In conclusion, TM28 treatment can enhance physiological and biochemical responses in wheat seedlings subjected to *Fp* stress and increase wheat resistance to pathogen infection by enhancing antioxidant defense activity. The effects of TM28 inoculation on *Fp* copy numbers in different parts of wheat are shown in Figure 5. Compared with the control, TM28 treatment significantly reduced the abundance of *Fp* in the rhizosphere soil, stem, and roots of Jimai 22 and Jimai 44. For Jimai 22, TM28 treatment reduced the abundance of *Fp* in the rhizosphere soil, stem, and roots by 36.31, 79.92, and 40.94% compared to control, respectively. For Jimai 44, TM28 treatment significantly reduced the abundances from 2.99 × 10^8^ copies g ^−1^ to 5.56 × 10^7^ copies g ^−1^ and 5.4 × 10^7^ copies g ^−1^ to 2.3 × 10^7^ copies g ^−1^ in wheat stem and root, respectively.

### 3.5 Transcriptome of *Fusarium pseudograminearum* co-cultured with TM28

**Figure 6:**
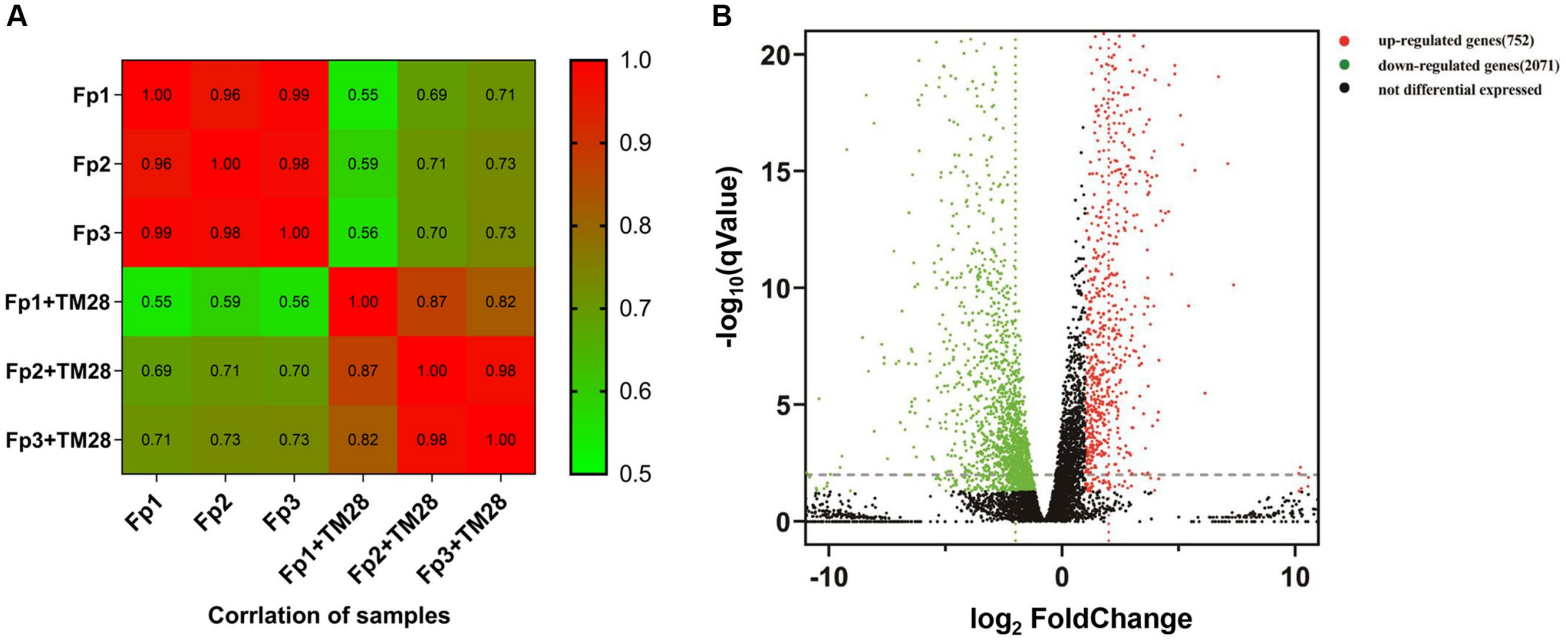
**(A)** Correlation analysis between samples. The color block represents the correlation index value, the greener color means a lower correlation index between the samples, and the red color a higher correlation index. **(B)** Volcano map of differentially expressed genes (DEGs) in *F. pseudograminearum* treated with sterile fermentation filtrate of *T. muroii* TM28. The y-axis corresponds to the mean expression value of -log_10_ (qValue), and the x-axis represents log_2_ (fold change). Red dots represent up-regulated DEGs, and green dots represent downregulated DEGs. The black dots indicate genes that were not differentially expressed.

A total of 42.5 Gb clean reads were obtained after filtering with a number of 46.2 Gb raw reads from the six samples of *F. pseudograminearum* under controls (Fp) and treatments (Fp + TM28). The average clean reads of the control and treatment groups were 48,546,415 and 50,514,798 reads, respectively. In the control group, the range of Q20 was 98.60 to 98.72% (Supplementary Table S2), the value of Q30 was higher than 94.96%, and GC content was stable between 52.67 and 52.82%. In the treatment group, the average value of Q20 and Q30 values were 98.73 and 95.39%, respectively, and GC content ranged from 53.27 to 53.53%. Of all the clean reads, 84.53–87.32% were successfully mapped to the *F. pseudograminearum* genome, and 84.41–86.88% were uniquely mapped (Supplementary Table S3). These results showed that the quality of the data generated by RNA-seq was sufficient for further investigation using subsequent bioinformatics. The correlation coefficients of the gene expression level between biological replicates in the control ranged from 0.82 to 0.98. Moreover, the correlation coefficients between the biological replicates in the treatment group ranged from 0.96 to 0.99. The high repeatability between replicates indicated that the RNA-seq data were reliable and could be used for further analyses (Figure 6A).

A total of 11,569 expressed genes, including 10,763 well-known genes and 806 new genes, were identified. An absolute fold change ≥2 and *p* < 0.05 were used to define differentially expressed genes (DEGs). A total of 2,823 significant DEGs were identified, including 2071 down-regulated genes and 752 up-regulated genes (Figure 6B). Accounting for 8.86% of the total DEGs, 250 genes had expression differences greater than 5-fold, comprising 42 up-regulated and 208 downregulated (Supplementary Table S4).

### 3.6 GO and KEGG enrichment analysis of the differential expressed genes

**Figure 7:**
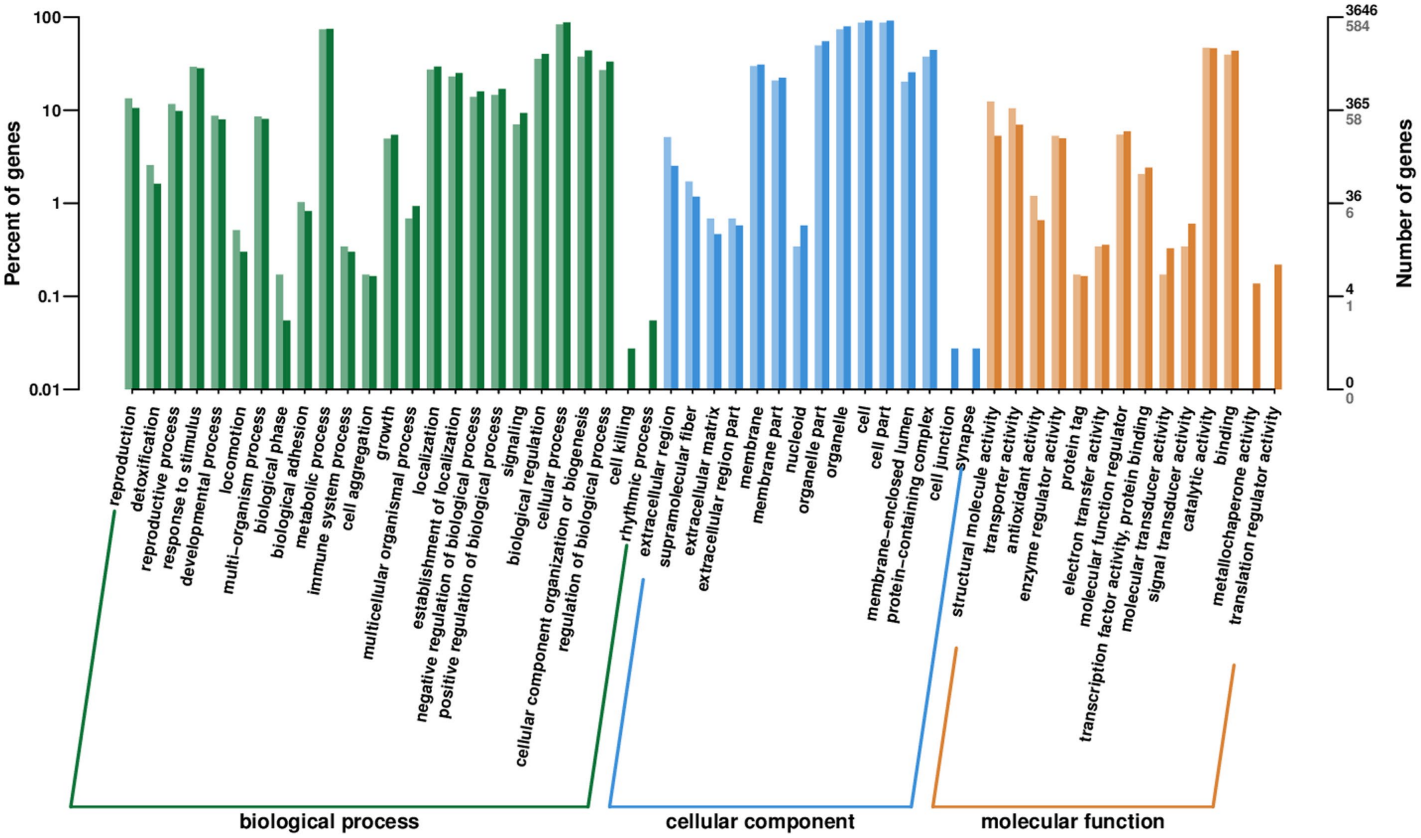
Gene Ontology (GO) enrichment analysis of the differentially expressed genes (DEGs) of *F. pseudograminearum* treated with sterile fermentation filtrate of *T. muroii* TM28. The horizontal axis represents the enriched terms, and the vertical axis represents the number of genes within that term (right) and the percentage of the total number of annotated genes (left). Different colors represent different terms. Light colors represent DEGs and dark colors represent all genes.

A total of 5,630 DEGs were classified into 54 GO terms, including biological processes (BP), cellular components (CC), and molecular function (MF). For BP, 2482 genes were differentially expressed, and the top three terms were cellular process (19.62%, 299 downregulated), metabolic process (17.41%, 262 downregulated), and cellular component organization or biogenesis (8.82%, 132 downregulated). There were 2,423 genes involved in 13 terms of CC pathways, including cell, cell part, organelle, organelle part, protein-containing complex, membrane, and membrane part, of which 1,496 DEGs were downregulated. There were 725 genes involved in 11 MF pathways, including catalytic activity, binding, transporter activity, and structural molecule activity, of which 437 were downregulated (Figure 7). For the downregulated DEGs, the number of genes enriched in organic substance biosynthetic process, organonitrogen compound metabolic processes, and cellular biosynthetic processes was higher, while organic acid and carboxylic acid catabolic processes were most significantly enriched. Among the up-regulated DEGs, the three functions of translation, cytoplasmic translation, and ribosomes were the most significantly enriched.

We performed a Kyoto Encyclopedia of Genes and Genomes (KEGG) enrichment analysis to further investigate the potential functions of the identified DEGs. There was a total of 2,229 DEGs classified into 205 pathways and five functional groups. The majority of DEGs were enriched in metabolic processes; 293 were associated with carbohydrate metabolism, amino acid metabolites, and lipid metabolism, and 66.6% were down-regulated.

**Figure 8:**
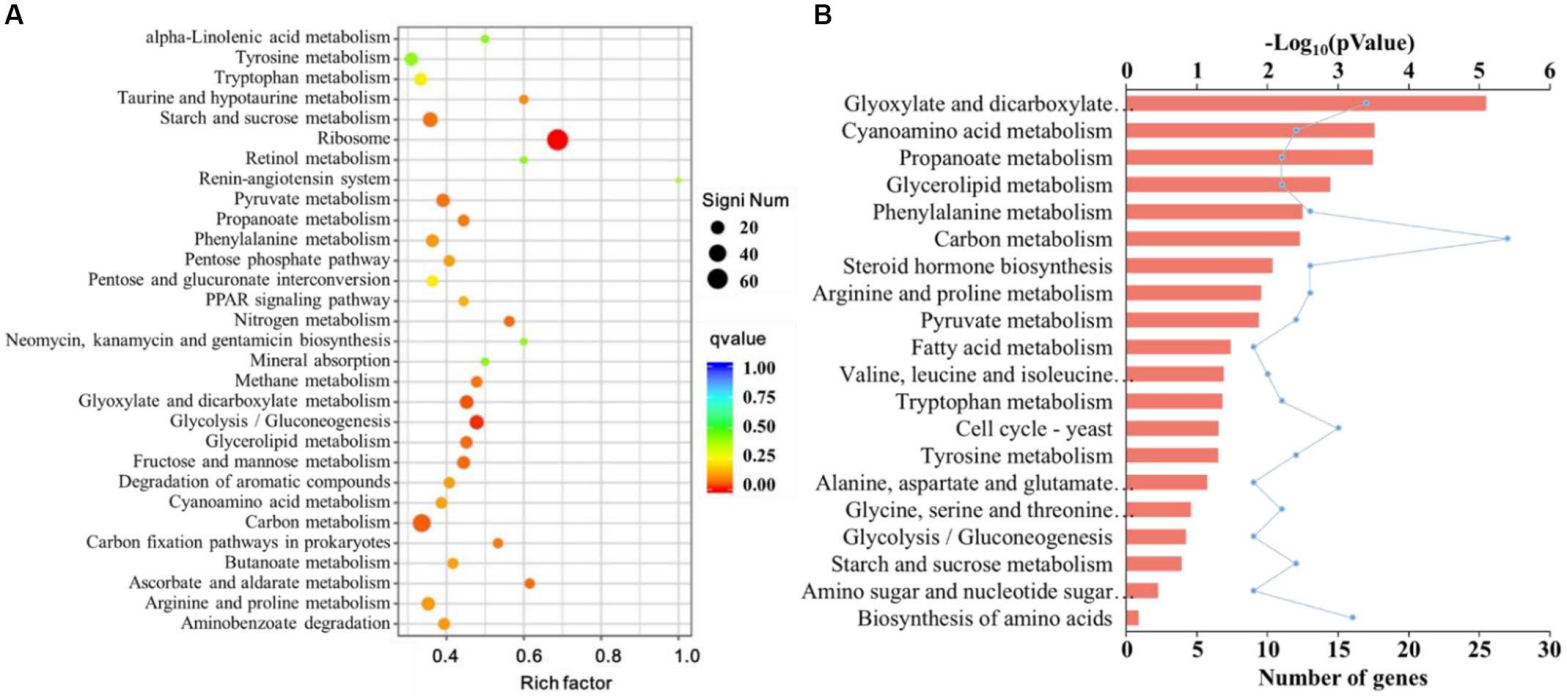
**(A)** Kyoto Encyclopedia of Genes and Genomes (KEGG) enrichment analysis of the differentially expressed genes (DEGs) of *F. pseudograminearum* treated with sterile fermentation filtrate of *T. muroii* TM28. The horizontal axis represents the ratio of the number of DEGs annotated to this pathway to the total number of DEGs, and the vertical axis represents the pathway classification. The dots from blue to red indicate the *p*-values from large to small, and the size of the dots indicates the number of DEGs annotated to this pathway. **(B)** The top 20 KEGG pathway analysis of downregulated DEGs in *F. pseudograminearum* treated with sterile fermentation filtrate of *T. muroii* TM28. The ordinate represents the KEGG term, and the upper abscissa represents the significance level of enrichment, corresponding to -Log_10_ (pValue) in the legend. The lower abscissa indicated the number of genes.

The top 10 KEGG pathways with the most DEGs were ribosome (66, all upregulated), carbon metabolism (41 with 14 upregulated and 27 downregulated), biosynthesis of amino acids (27 with 11 upregulated and 16 downregulated), starch and sucrose metabolism (24 with 12 upregulated and 12 downregulated), glycolysis/gluconeogenesis (22 with 13 upregulated and 9 downregulated), glyoxylate and dicarboxylate metabolism (19 with 2 upregulated and 17 downregulated), pyruvate metabolism (18 with 6 upregulated and 12 downregulated), arginine and proline metabolism (18 with 5 upregulated and 13 downregulated), tyrosine metabolism (17 with 5 upregulated and 12 downregulated), and amino sugar and nucleotide sugar metabolism (17 with 8 upregulated and 9 downregulated). Nine of the ten significant DEGs annotated to amino acid metabolic pathways (ko01212) were downregulated (Figure 8A).

Among the downregulated genes, 16 pathways were related to metabolism, and were mainly enriched in glyoxylate and dicarboxylate metabolism, cyanoamino acid metabolism, propanoate metabolism, glycerolipid metabolism, phenylalanine metabolism, and carbon metabolism (Figure 8B). Steroid biosynthesis, pyruvate and fatty acid metabolism were also enriched.

### 3.7 Analysis of key DEGs and pathways involved in *Fusarium pseudograminearum* response to TM28 antagonism

The Pfam database (www.ebi.ac.uk/interpro) was used for the functional annotation of DEGs, and genes related to *Fp* cell wall synthesis, cell membrane synthesis, and energy metabolism were annotated and summarized.

#### 3.7.1 The key DEGs related to cell wall and cell membrane synthesis of *Fusarium pseudograminearum*

For cell wall synthesis, chitin synthase CHS (HYE68_007183, HYE68_005139), beta-glucosidase (HYE68_001336), and 1, 3-beta-glucanosyltransferasen (HYE68_010124) were all significantly downregulated (Table 2).

**Table 2:** Unigenes related to cell wall and cell membrane synthesis of *F. pseudograminearum*.

Gene Name	Gene Annotation (Log2 FC)	Pass name (Route number)
HYE68_007183	Chitin synthase, CHS (−11.32*)	Amino sugar and nucleotide sugar metabolism (Map 00520)
HYE68_005139	Chitin synthase, CHS (−2.53*)	Amino sugar and nucleotide sugar metabolism (Map 00520)
HYE68_001336	beta-glucosidase (−3.99*)	Starch and sucrose metabolism (Map 00500)
HYE68_010124	1,3-beta-glucanosyltransferase (−1.49)	Starch and sucrose metabolism (Map 00500)
HYE68_009412	Fatty acid synthase, FAS1(−2.08*)	Fatty acid metabolism (Map 01212)
HYE68_009411	Fatty acid synthase, FAS2(−2.03*)	Fatty acid metabolism (Map 01212)
HYE68_005110	acetyl-CoA carboxylase (−2.2*)	Fatty acid metabolism (Map 01212)
HYE68_009836	Sterol 24-C-methyltransferase, ERG6(−4.66*)	Steroid biosynthesis (Map 00100)
HYE68_007214	Fatty acid hydroxylase (4.82*)	Fatty acid degradation (Map 00071)
HYE68_002214	Sterol 14α-Demethylase, CYP51(−4.66*)	Steroid biosynthesis (Map 00100)
HYE68_005067	Cycloeucalenol cycloisomerase (−12.09*)	Steroid biosynthesis (Map 00100)

Asterisks indicate significant differences between groups samples according to ANOVA and Tukey’s test. (*p* < 0.05).

For cell membrane synthesis, TM28 significantly downregulated *FAS1* (HYE68_009412) and *FAS2* (HYE68_009411), which were involved in phospholipid biosynthesis. Acetyl-CoA carboxylase (HYE68_005110), which involved in fatty acid synthesis and extension, was downregulated. In contrast, the expression of the (HYE68_007124) gene, which has fatty acid hydroxylase activity and catalyzes the formation of α-hydroxy fatty acids from fatty acids, was significantly up-regulated by 4.82-fold (Table 2). In the steroid biosynthesis pathway (map00100), CYP51 (HYE68_002214) and ERG6 (HYE68_009836) related to ergosterol synthesis were significantly downregulated. The expression of cycloeucalenol cycloisomerase CPI1 (HYE68_005067), a key enzyme in the steroid biosynthesis pathway, was downregulated by 12.09-fold (Table 2).

#### 3.7.2 The key pathways involved in *Fusarium pseudograminearum* response to TM28 antagonism

Combined with GO enrichment and KEGG enrichment, we found that TM28 affected carbohydrate metabolism, amino acid metabolism, and lipid metabolism in *F. pseudograminearum*, and the DEGs were screened from all the differentially expressed genes related to glycolysis/gluconeogenesis, glyoxylate and dicarboxylate metabolism, pyruvate metabolism, TCA cycle, and pentose phosphate pathway (Table 3).

**Table 3:** Unigenes related to glucose and energy metabolism and transportation of *F. pseudograminearum.*

Gene Name	Gene Annotation (Log_2_ FC)	Pass name (Route number)
HYE68_000608	Phosphoenolpyruvate carboxykinase, PEPCK (−3.66*)	Citrate cycle (TCA cycle)(Map 00020)
HYE68_008522	Isocitrate dehydrogenase, IDH (−1.94)	Carbon metabolism (Map 01200)
HYE68_005552	Acetyl-coenzyme A synthase (−5.49*)	Pyruvate metabolism (Map 00620)
HYE68_005398	Citrate synthase (−2.14*)	Citrate cycle (TCA cycle)(Map 00020)
HYE68_004615	Pyruvate carboxylase (−1.76)	Citrate cycle (TCA cycle)(Map 00020)
HYE68_004002	Hexokinase, HK (3.34*)	Glycolysis/Gluconeogenesis (Map 00010)
HYE68_003319	Phosphofructokinase, PFK (1.48)	Glycolysis/Gluconeogenesis (Map 00010)
HYE68_003135	Fructose-1,6-bisphosphatase, FBP (−1.88)	Glycolysis/Gluconeogenesis (Map 00010)
HYE68_001213	Hexokinase, HK (3.99*)	Glycolysis/Gluconeogenesis (Map 00010)
HYE68_008621	Phosphoenolpyruvate synthase, PEPs (−2.14*)	Glycolysis/Gluconeogenesis (Map 00010)
HYE68_008620	Lactate dehydrogenase, LDH (−2.43*)	Glycolysis/Gluconeogenesis (Map 00010)
HYE68_006936	ATP-binding cassette transporter, ABCB1(−4.96*)	ABC transporters (Map 02010)
HYE68_004175	ATP-binding cassette transporter, ABCB1(−2.29*)	ABC transporters (Map 02010)
HYE68_002077	Glutathione S-transferase (−12.41*)	Glutathione metabolism (Map 00480)
HYE68_002746	MFS profile domain-containing protein (−14.78*)	Plasma membrane (GO:0005886)

Asterisks indicate significant differences between groups samples according to ANOVA and Tukey’s test. (*p* < 0.05).

After TM28 filtrate treatment, phosphoenolpyruvate carboxykinase PEPCK (HYE68_000608), phosphoenolpyruvate synthase PEPs (HYE68_008621), fructose-1,6-bisphosphatase FBPase (HYE68_003135), and LDH (HYE68_008620) were significantly downregulated, whereas HK (HYE68_004002, HYE68_001213), PFK (HYE68_003319), and PK (HYE68_005136) were upregulated (Table 3). IDH1 (HYE68_008522), citrate synthase (HYE68_005398), and pyruvate carboxylase (HYE68_004615), which are involved in the TCA cycle, were downregulated. Acetyl-coA synthase (HYE68_005552), the central enzyme of metabolism in eukaryotes, was significantly downregulated by 5.49-fold.

Two genes encoding ABC transporters (HYE68_006936 and, HYE68_004175) were significantly down-regulated during the removal and transportation of toxins and toxic compounds. Glutathione S-transferase (HYE68_002077), which is related to glutathione metabolism, was significantly downregulated by 12.41-fold. The MFS domain-containing protein (HYE68_002746) was significantly downregulated by 14.78 times.

### 3.8 RT-PCR of *Fusarium pseudograminearum* genes co-cultured with TM28

To test the accuracy of the transcriptome sequencing results, 20 DEGs involved in cell wall synthesis, cell membrane synthesis, and metabolism were selected for qRT-PCR validation, including 4 upregulated and 16 downregulated genes. The results (Supplementary Figure S1) revealed that the expression levels of the selected genes were consistent with the transcriptome results, indicating that the transcriptome sequencing results were reliable.

## 4 Discussion

FCR is a significant disease in China that poses a significant risk to the production of wheat. It has been demonstrated that antagonistic microorganisms not only inhibit the growth and development of pathogens by producing antibiotics, toxins, and extracellular degrading enzymes, but they can also stimulate the defensive response of plants, thereby increasing their resistance to pathogens (Mon et al., 2021). It was previously reported that *Bacillus velezensis* YB-185 had strong inhibitory effects on *Fp* mycelium growth and spore germination and significantly reduced stem basal rot in wheat (Zhang et al., 2022). The inhibition rate of PDA medium containing 20 and 66% of *B. siamensis* YB-1631 fermentation filtrate on *Fp* spore germination was 84.14 and 92.23%, respectively (Dong et al., 2023) In the present study, the TM28 strain of *T. muroii* proved to be a promising antagonistic microorganism for the control of wheat FCR caused by *Fp*.

In this study, TM28 showed strong antifungal activity against *Fp* with an inhibition rate of 87.8% (Figure 1A). The sterile fermentation filtrate of TM28 inhibited mycelial growth (Figure 1C) and conidial germination (Figure 2) of *Fp* by destroying the cell wall and cell membrane structures. Under greenhouse conditions, TM28 treatment significantly increased the biomass of wheat plants in the presence of pathogen *Fp* (Table 1). Inoculation with TM28 ameliorated the negative effects of *Fp*, reducing disease severity and pathogen abundance in the rhizosphere soil, root and stem base of wheat (Figure 5). The results demonstrated a significant increase in antioxidant defense activity in TM28-inoculated wheat plants compared to pathogen-infected plants (Figure 4). At the seedling stage, both Jimai22 and Jimai44 seeds treated with TM28 demonstrated effective control of FCR, with 58.49 and 51.69% inhibition, respectively (Table 1). Previous studies have shown that *Talaromyces* spp. are widely present in the soil and air, and some strains can be used to control plant diseases (Dethoup et al., 2018). *T. flavus*, which was isolated from the rhizosphere soil of tomatoes, had a significant effect on tomato blight caused by *Verticillium dahlia* (Naraghi et al., 2010b). The Q2 strain of *T. purpureogenus* can inhibit the growth of 12 plant pathogens and prevent soil-borne diseases such as bitter melon wilt, tobacco black shin disease, and potato stem rot under greenhouse conditions (Tian et al., 2020). These results indicate that *T. muroii* TM28 can effectively inhibit the growth of *Fp*. To determine the antagonistic mechanism of TM28, we examined the gene expression levels of *Fp* using high-throughput sequencing of the transcriptome.

In the present study, we found that TM28 directly inhibited the growth of *Fp* and produced a clear inhibition zone in the co-culture plate (Figure 1A). In addition, the sterile fermentation filtrate of TM28 inhibited the germination of *Fp* spores and induced mycelium swelling and deformation. This was likely a result of TM28’s production of numerous cell-wall-degrading enzymes and other catalytic activities. Previous studies have shown that *Talaromyces* spp. contains rich and diverse genes encoding proteases (Haggag et al., 2006), secondary metabolite biosynthetic enzymes (Wang et al., 2020), and fungal cell wall-degrading enzymes (Aggarwal et al., 2023). The cell wall is essential for fungal viability, morphogenesis, and pathogenesis (Kim and Lee, 2012), and degradation of the cell walls of plant pathogens is important for antagonistic microorganisms (Karthik et al., 2017). *T. flavus* was capable of producing two chitinases, 41 kDa and 32 kDa, which degraded the cell wall of *Verticillium dahliae* and *Rhizoctonia solani*, and inhibited spore germination and germ tube elongation of *Alternaria alternate* (Li et al., 2005).

Chitin and β-(1,3) – glucan are key components of the *Fp* cell wall and are synthesized by chitin synthase and β-(1,3)-glucan synthase, respectively (Lenardon et al., 2010). Chitin is important for cell division and virulence (Plaza et al., 2020). Previous studies have shown that 1,3-beta-glucan can alter the morphology of fungal mycelia, promote mycelial extension and growth, and maintain mycelial stability (Ruiz-Herrera and Ortiz-Castellanos, 2019; Cow and Lenardon, 2023), Deletion of the 1,3-beta-glucanosyltransferase gene results in changes in cell wall morphology and abnormal conidia (Zhao et al., 2014). In the present study, we found that the expression of chitin synthase (HYE68_007183 and HYE68_005139) was significantly downregulated by 11.32-fold and 2.53-fold, respectively, and that the expression of beta-glucosidase (HYE68_001336) was significantly downregulated by 3.99-fold (Table 2). Additionally, 1,3-beta-glucanosyltransferase (HYE68_010124), which is involved in the elongation of 1,3-beta-glucan significantly downregulated. We hypothesized that TM28 affects pathogen growth by downregulating the expression of genes involved in *Fp* cell wall synthesis.

TM28 also alters the expression of genes related to fungal cell membrane biosynthesis. In this study, several DEGs, including *FAS1* (HYE68_009412), *FAS2* (HYE68_009411), and ACC (HYE68_005110), which are involved in the fatty acid synthesis pathway, were significantly downregulated (Table 2). In contrast, cytochrome P450 reductase *cypd* (HYE68_007214), which is involved in the fatty acid degradation pathway, was significantly upregulated by 4.82-fold (Table 2). Previous studies have shown that the *Magnaporthe oryzae* mutant strain *∆fas1*, which is a deletion of the *FAS1* gene, has significantly lower mycelial dry weight and conidium production (Sangappillai and Nadarajah, 2020). Chayakulkeeree et al. (2007) found that the CFU of *C. neoformans* decreased 100-fold when *FAS1* or *FAS2* expression was downregulated. Ergosterol is another important component of fungal cell membranes that affects membrane fluidity and permeability (Volkman, 2003). Once ergosterol synthesis is blocked, it disrupts the structure and function of the cell membrane and inhibits fungal growth (Sousa-Lopes et al., 2004; Waki et al., 2008). In this study, we found that the ergosterol biosynthesis gene, *ERG6* (HYE68_009836), was significantly downregulated (Table 2). *DWF5* (HYE68_001781), *CYP51* (HYE68_002214), and cycloeucalenol cycloisomerase (HYE68_005067), which are involved in steroid synthesis, were significantly downregulated. The cell membrane plays an important role in the transport of osmoprotective compounds (Liu et al., 2018), and an increase in cell membrane permeability causes the leakage of intracellular substances and an increase in electrical conductivity, leading to cell death (Furuya et al., 2011; Wei et al., 2022). The present study demonstrated that the electrical conductivity of the *Fp* fermentation broth was significantly higher after TM28 treatment compared to the control (Figure 3A), suggesting that the metabolites produced by TM28 affected the membrane permeability of *Fp*. This hypothesis was further verified by microscopic observation of swelling and morphological irregularities in *Fp* mycelia and spores after TM28 treatment. This result was consistent with the effect of the antimicrobial protein of *B. amyloliquefaciens* HRH317 on *F. graminearum* observed by scanning electron microscopy (Qin, 2015).

Central carbohydrate metabolism, consisting of glycolysis, pentose phosphate pathway, and TCA cycle, converts nutrients into energy and precursors and is the most fundamental metabolic process for maintaining normal cell growth (Zhang Z. et al., 2020; Wu et al., 2023). In the present study, hexokinase HK and PFK (HYE68_004002, HYE68_001213), involved in glycolysis, were significantly upregulated (Table 3), accelerating the accumulation of glucose 6-phosphate and the conversion of β-1,6-2 phosphofructose (Granot et al., 2014; Yu et al., 2022). This result was consistent with the changes in HK and PFK activities in the *Fp* fermentation broth after TM28 treatment (Figures 3C,D). Meanwhile, the expression of three DEGs for acetyl-CoA synthetase, pyruvate carboxylase, and citrate synthase was downregulated. Consequently, oxaloacetic acid and acetyl-CoA cannot be converted into citric acid in time and enter the TCA cycle (Llamas-Ramirez et al., 2020; Tovilla-Coutino et al., 2020; Jezewski et al., 2021), ultimately affecting *Fp* metabolism and physiological balance. Phosphoenolpyruvate, as a crucial intermediate linking glycolysis and the TCA cycle, contributes to the amount of ATP required for cellular energy metabolism (Torresi et al., 2023). Oxaloacetate and pyruvate are catalyzed by PEPCK and PEPs to generate phosphoenolpyruvate, TM28 downregulates the expression of PEPCK, and PEPs further inhibit TCA cycling and ATP energy production in *Fp*.

The DEGs related to transportation in *Fp* were significantly downregulated, including two ABC transporter-related genes (HYE68_006936 and HYE68_004175) and GST (HYE68_002077) (Table 3). ABC transporters can eliminate toxins by transferring substrates across membranes (Rees et al., 2009) or guiding their secretion into the extracellular environment (Chen et al., 2021). GST is a detoxifying enzyme (Dasari et al., 2018) that was significantly downregulated in the present study. MFS transporters are more selective than ABC transporters in the efflux of toxin compounds (Lee et al., 2011) previous research has shown that the deletion of MFS and ABC transporters inhibits *FP* growth (Li et al., 2020). In the present study, the MFS transporter (HYE68_002746) was significantly downregulated, which inhibited the ability of *Fp* to extract toxic compounds, thereby reducing pathogenicity.

## 5 Conclusion

*Talaromyces muroii* TM28 has potent antagonistic activity *in vitro* against *F. pseudograminearum* by inhibiting conidial and hyphal growth. It stimulated the production of antioxidant enzymes in wheat and reduced the colonization of *Fp* in the rhizosphere soil, roots and the stem bases of wheat in pot experiments. Transcriptome sequencing analysis revealed that the differentially expressed genes related to cell wall and cell membrane synthesis of *Fp* were significantly downregulated under TM28 antagonism, and the culture filtrate of TM28 affected the pathways of fatty acid synthesis, steroid synthesis, glycolysis, and the TCA cycle, reducing the transport of toxins. We hypothesized that TM28 reduced the pathogenicity of *Fp* in wheat by disrupting the cell wall and cell membrane structures and by regulating the process of energy formation. However, a more comprehensive antagonistic mechanism of *Talaromyces* spp. requires further study. Overall, these results provide a foundation for future research into the biocontrol mechanisms and potential applications of *Talaromyces* spp.

## Data availability statement

The datasets presented in this study can be found in online repositories. The names of the repository/repositories and accession number(s) can be found in the article/Supplementary material.

## Author contributions

HaY: Data curation, Investigation, Writing – original draft, Writing – review & editing. SC: Data curation, Investigation, Writing – original draft. YaW: Conceptualization, Data curation, Investigation, Supervision, Writing – review & editing. HL: Data curation, Formal analysis, Writing – original draft. JH: Data curation, Funding acquisition, Resources, Writing – review & editing. KY: Data curation, Resources, Software, Writing – review & editing. YuW: Funding acquisition, Software, Writing – review & editing. ZZ: Formal analysis, Software, Validation, Writing – review & editing. JL: Funding acquisition, Project administration, Visualization, Writing – review & editing. YiW: Data curation, Investigation, Validation, Writing – review & editing. HeY: Conceptualization, Methodology, Validation, Writing – review & editing.

## Footnotes

^1^https://www.ncbi.nlm.nih.gov/genome/14399?genome_assembly_id=1560984
