# Supplementary material for: Antagonistic effects of Talaromyces muroii TM28 against Fusarium crown rot of wheat caused by Fusarium pseudograminearum
Source: Front Microbiol. 2024 Jan 3;14:1292885. doi: 10.3389/fmicb.2023.1292885 (PMC10791928; doi:10.3389/fmicb.2023.1292885)
Supplement: Supplementary file 1 [file Presentation_1.PPTX]

## Slide 1
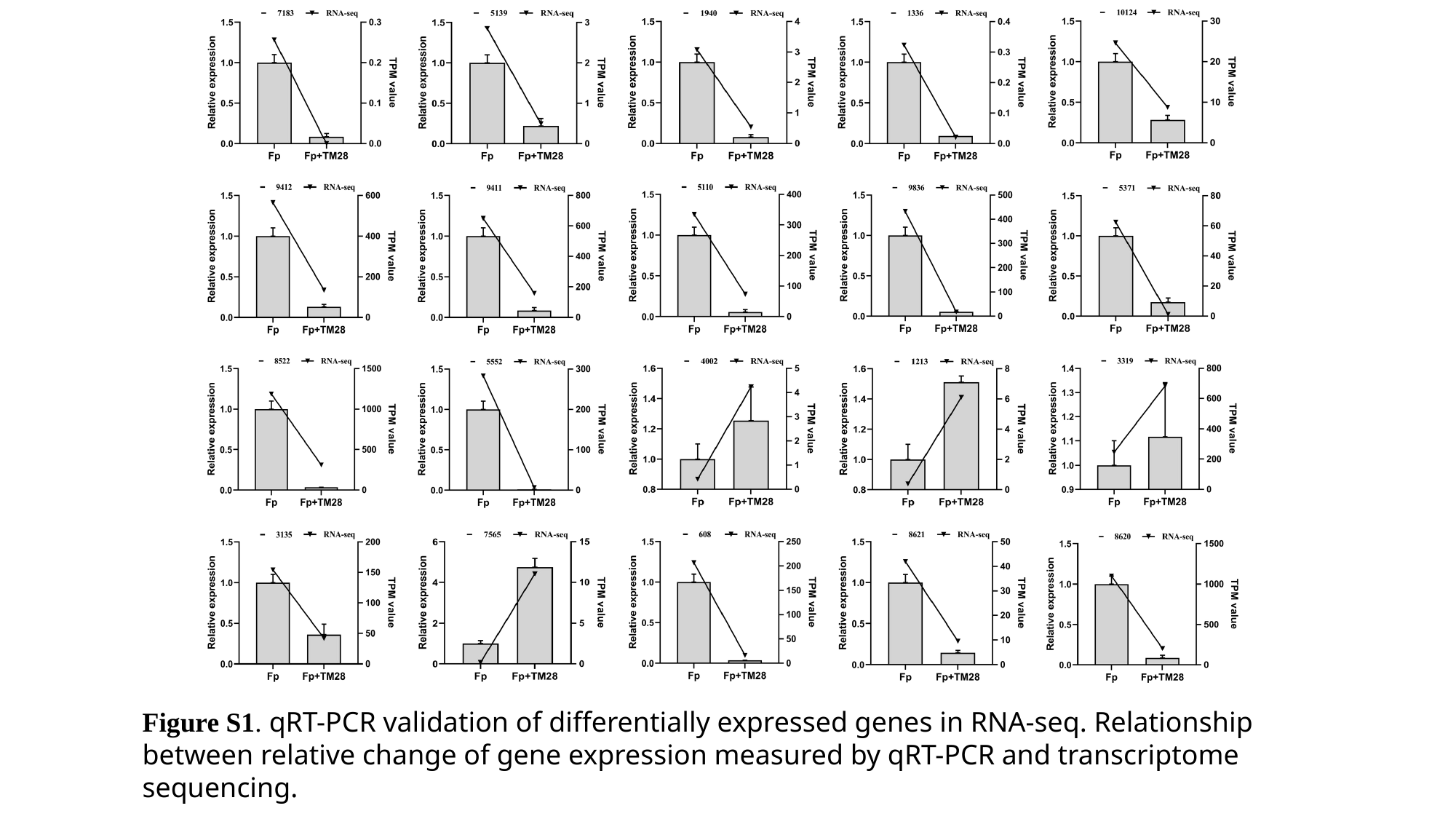

Figure S1. qRT-PCR validation of differentially expressed genes in RNA-seq. Relationship between relative change of gene expression measured by qRT-PCR and transcriptome sequencing.
